# Automated Discrimination of Appearance Quality Grade of Mushroom (*Stropharia rugoso-annulata*) Using Computer Vision-Based Air-Blown System

**DOI:** 10.3390/s25144482

**Published:** 2025-07-18

**Authors:** Meng Lv, Lei Kong, Qi-Yuan Zhang, Wen-Hao Su

**Affiliations:** College of Engineering, China Agricultural University, 17 Qinghua East Road, Haidian, Beijing 100083, China; lv.meng@cau.edu.cn (M.L.); s20243071636@cau.edu.cn (L.K.); s20233071522@cau.edu.cn (Q.-Y.Z.)

**Keywords:** deep learning, OpenCV, *Stropharia rugoso-annulata*, grading system, motion trajectory

## Abstract

The mushroom *Stropharia rugoso-annulata* is one of the most popular varieties in the international market because it is highly nutritious and has a delicious flavor. However, grading is still performed manually, leading to inconsistent grading standards and low efficiency. In this study, deep learning and computer vision techniques were used to develop an automated air-blown grading system for classifying this mushroom into three quality grades. The system consisted of a classification module and a grading module. In the classification module, the cap and stalk regions were extracted using the YOLOv8-seg algorithm, then post-processed using OpenCV based on quantitative grading indexes, forming the proposed SegGrade algorithm. In the grading module, an air-blown grading system with an automatic feeding unit was developed in combination with the SegGrade algorithm. The experimental results show that for 150 randomly selected mushrooms, the trained YOLOv8-seg algorithm achieved an accuracy of 99.5% in segmenting the cap and stalk regions, while the SegGrade algorithm achieved an accuracy of 94.67%. Furthermore, the system ultimately achieved an average grading accuracy of 80.66% and maintained the integrity of the mushrooms. This system can be further expanded according to production needs, improving sorting efficiency and meeting market demands.

## 1. Introduction

The mushroom *Stropharia rugoso-annulata* is recognized as one of the top ten cultivated mushroom varieties in the global market due to its rich nutritional value and firm texture [1,2]. This mushroom is commercially graded based on appearance quality, with distinct differences in nutritional content and market value observed across grades. Current grading processes rely heavily on manual sorting, resulting in subjective standards, inconsistent accuracy, and low efficiency [3,4]. Therefore, it is crucial to develop a fast and accurate automatic mushroom classification system.

In recent years, deep learning has achieved remarkable progress in machine vision tasks such as detection, classification, and segmentation through powerful and efficient feature extraction [5,6,7,8,9,10]. YOLO-series models have also been applied in mushroom grading. Farshbaf Aghajani et al. [11] achieved 96% accuracy classifying frozen mushrooms with YOLOv5-assisted ultrasound, while Liu et al. [12] proposed the APHS-YOLO model for *Stropharia rugoso-annulata*, reducing the parameters by 57.8% with over 100 FPS. Liu et al. [13] and Cong et al. [14] made significant progress in shiitake classification by improving the YOLOX and YOLOv3 models, attaining an mAP of 99.96% and improving the mAP by 2.04%. Wu et al. [15] further fused YOLOv5 and PSPNet to propose an algorithm for size-graded detection of antlers, which improved the pixel accuracy to 96.35%. Despite these advancements, key challenges remain, such as the reliance on qualitative grading criteria, a lack of standardization, and the inability to assess morphological proportions effectively. For the first time, our study proposed the SegGrade algorithm, introducing quantitative indicators (the RDHP and RLDS) to the field of mushroom grading. This algorithm adopts the method of “segmentation first and then grading”, which can more accurately evaluate mushroom morphology and can achieve efficient and accurate grading.

Effective grading requires robust algorithms paired with reliable hardware systems [16,17]. Gan et al. [18] developed a machine vision-based air-blown system for tea leaves, achieving 25 kg/h without damaging them. Zhang and Chuah [19] designed a rotating circular conveyor system that directs mangosteens into designated grading slots. The above methods have not yet been applied to mushroom grading. Mohi-Alden et al. [20] and Nakaguchi et al. [21] used pneumatic actuators to sort peppers and quail eggs, achieving sorting efficiencies of 3000 sweet peppers per hour and 797 quail eggs per hour. However, such mechanisms are unsuitable for mushrooms due to their fragility. Wang et al. [22] designed an automatic white mushroom sorter that operates at 102.41 mushrooms per minute, but it lacks a feeding mechanism. Many current systems still rely on manual feeding, increasing the labor intensity and limiting automation [23,24,25]. Therefore, it is crucial to minimize physical damage during the grading process and develop a fully automatic grading device. Our research has developed a mushroom-grading device that is fully automatic from feeding to grading. It can complete the grading of 20 mushrooms within one minute and does not cause any damage to the mushrooms during the process.

The main contents of this study are as follows: (1) constructing datasets of the different grades of the mushroom; (2) proposing suitable value ranges for the grading criteria for the mushroom based on quantitative grading indexes (the RDHP and RLDS); (3) comparing six segmentation models and selecting the YOLOv8-seg model, which better balances accuracy and speed in cap and stalk segmentation; (4) proposing the SegGrade algorithm for the mushroom based on the idea of segmentation followed by grading; and (5) developing an efficient, non-destructive air-blown grading system. To the best of our knowledge, this is the first time that a SegGrade algorithm based on quantitative indexes has been proposed for crops. The proposed grading method is also applicable when grading crops of the same shape.

## 2. Materials and Methods

### 2.1. System Structure and Principle

**Figure 1:**
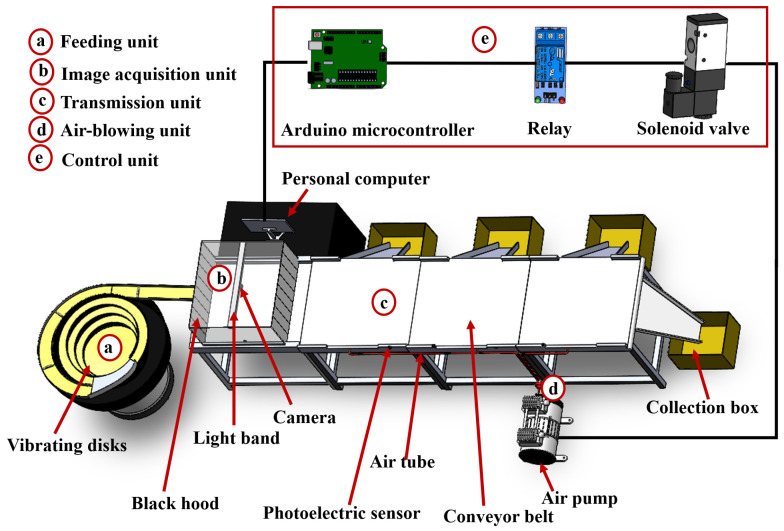
The designed automatic mushroom-grading system.

The designed automatic mushroom-grading system is illustrated in Figure 1. This system consists of five main components: a feeding unit, an image acquisition unit, a transmission unit, an air-blown unit, and a control unit. In the automatic grading system, an air-blown method is utilized to achieve non-destructive grading of mushrooms into three grades. When the mushrooms are discharged from the feeding unit, they pass through the first-grade, second-grade, and third-grade air tubes in turn and are eventually blown down into the corresponding collection boxes. When broken mushrooms are recognized, the grading unit is not activated, and they fall into the terminal collection box with the transmission unit.

The main components of the five parts of the automatic mushroom-grading system are also shown in Figure 1. The feeding unit consists of a vibrating disk, which enables a large quantity of mushrooms to fall on the conveyor belt in a single state in an orderly and even manner. The image acquisition unit, consisting of an industrial camera, a PC, a light band, a light bar, and a black hood, enables continuous image capture and transmission to the personal computer (PC) for grade recognition. The control unit is composed of an Arduino microcontroller, relays, photoelectric sensors, and solenoid valves. The Arduino microcontroller receives the grade information from the PC and controls the photoelectric sensors, relays, and solenoid valves to complete the air-blown grading. The air-blown unit mainly consists of an air pump, air tubes, and collection boxes. The control unit directs the air pump to open so that the corresponding air tube opens, ensuring that the mushrooms fall into the corresponding collection box.

During grading, multiple edible mushrooms are poured onto the vibrating disk and subsequently aligned in a single file on the conveyor belt. Grading is subsequently performed by activating the air pump through the coordinated operation of the air-blown unit and control unit. Specifically, individual mushrooms from the vibrating disk are transferred onto the conveyor belt to be fed to the image acquisition unit and photographed. The images are transferred to the PC via a universal serial bus (USB) cable. The PC uses the grading model to output a grading result and transmits the grade information to the Arduino microcontroller. When the photoelectric sensor detects the mushroom, the Arduino microcontroller activates the solenoid valve and the air pump opens to blow the mushroom into the collection box, thus completing the grading process.

In the feeding unit, a damage rate experiment was conducted to ensure that mushrooms are not damaged during vibratory conveying and to optimize the parameters of the vibratory feeder. In this experiment, 30 large mushrooms were randomly placed into the system, and tests were performed using various frequencies. The conveying time and the number of damaged mushrooms were recorded. The experiment consisted of three phases. In the coarse screening phase, six frequency values ranging from 50 to 75 Hz were tested at 5 Hz intervals, with each frequency repeated 10 times, to identify a safe frequency range. The results showed that higher frequencies led to shorter conveying times and significantly improved efficiency. Notably, at 60 Hz, no damage occurred, indicating favorable performance. In the fine-tuning phase, frequencies were further tested at 2 Hz intervals. It was found that mushrooms remained undamaged at 60 Hz, while a damage rate of 6.66% was observed at 62 Hz. These findings indicate that frequencies above a certain threshold may exceed the structural tolerance of mushrooms, leading to mechanical damage. Therefore, considering both efficiency and product integrity, 60 Hz was determined to be the optimal vibration frequency under the condition of zero damage.

**Figure 2:**
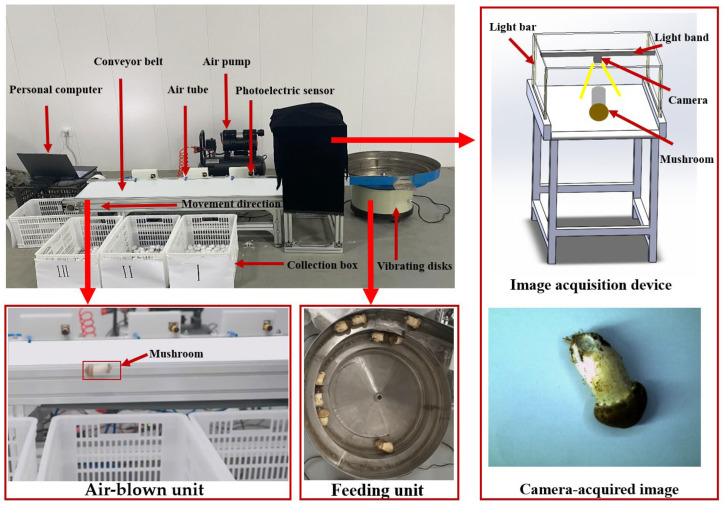
The implemented automatic mushroom-grading system.

In the image acquisition unit, the obtained image was uploaded to the laptop (Windows 11). The laptop executed the SegGrade algorithm for grade recognition, which was implemented in Python v3.9. In the air-blown unit, the mushroom trajectory was analyzed to adjust the positions of the collection boxes accordingly. In the control unit, the Arduino microcontroller received grade information from the laptop via USB. It also obtained sensor signals through pins and controlled the solenoid valve to complete the air-blown grading. The appearance and details of the constructed automatic mushroom-grading system are shown in Figure 2 (More details can be found in the Supplementary Materials).

### 2.2. Image Acquisition and Pre-Processing

To precisely extract segmented images of the caps and stalks of the mushrooms, many sample images needed to be acquired for training and testing using deep learning techniques [26]. In this study, images of mushrooms were captured at the experimental base in Dahuashan Town, Pinggu District, Beijing, China. Images were taken during the spring and autumn mushroom picking seasons to capture morphological differences across seasons. An MV-UBD130C industrial camera and a Huawei P60 smartphone were used to capture images. The image resolution of the camera was 1280 × 960, and the resolution of the smartphone was 2700 × 1220. To ensure no distortion when accurately photographing a mushroom, a photographic distance of 40 cm was always maintained between the photography equipment and the mushroom.

**Figure 3:**
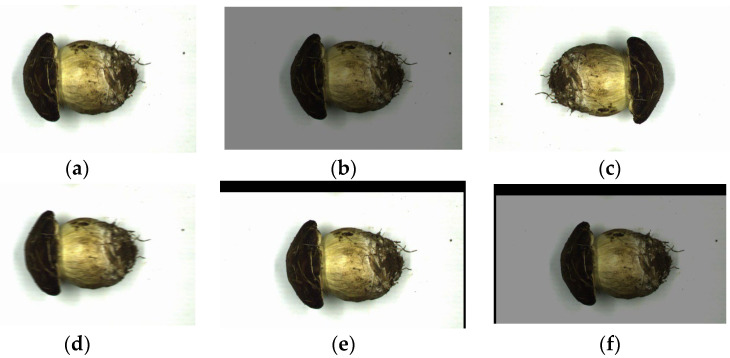
Image enhancement. (**a**) Original image. (**b**) Dark-light image. (**c**) Rotated image. (**d**) Image with Gaussian blurring. (**e**) Panned image. (**f**) Multifusion image.

In total, 500 images were taken under the same background and environmental conditions, and 336 high-quality original images were selected for this study. The cap and stalk parts of the mushrooms were manually labeled using the Labelimg tool to obtain the label files used for the experiment. Subsequently, the images and label files were randomly split into a test set, training set, and validation set with a ratio of 1:3:1, respectively. Too few images can easily lead to overfitting of a model during training [27]. To prevent model overfitting, 1680 images were obtained using the image enhancement process, and the final dataset construction is shown in Table 1. The image enhancement techniques used included rotation, panning, dark light, Gaussian blurring, and various fusions (as shown in Figure 3). Rotation and panning simulated the natural state of mushrooms on the conveyor belt; dark light addressed the uneven lighting problem that may occur in application scenarios; Gaussian blurring simulated the characteristics of moving targets captured by the camera; and the various fusions further improved the diversity and robustness of the data.

### 2.3. Development of Grading Criteria

**Figure 4:**
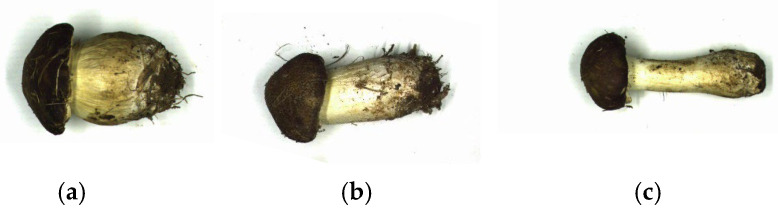
Appearance of mushrooms of different grades. (**a**) First-grade mushroom. (**b**) Second-grade mushroom. (**c**) Third-grade mushroom.

In contrast to previous grading criteria based on appearance characteristics such as color, shape, and ripeness, this research used quantitative grading indicators to grade mushrooms based on morphological qualities. Images of first-grade, second-grade, and third-grade mushrooms are shown in Figure 4, and the corresponding grading criteria were formulated according to their morphological appearance (as shown in Table 2). The grading criteria proposed in this study were developed by investigating the grading of these mushrooms by local enterprises in Dahuashan Town, Pinggu District, Beijing, China, and combining them with the local grading standard documents in Langfang, Hebei, China.

This study selected the ratio of diameter to height of the cap (RDHP) and the ratio of length to diameter of the stalk (RLDS) as mushroom-grading indicators. The mushrooms were graded according to the ranges to which the grading indexes belonged, and when the range of the RDHP of the mushrooms did not correspond to the range of the RLDS, the grades were classified according to the range of the RLDS.

The RDHP was defined as follows:

(1) $$\mathrm{RDHP}=\frac{D_{1}}{H}$$

where $D_{1}$ denotes the diameter of the cap and H represents the height of the cap.

The RLDS was defined as follows:

(2) $$\mathrm{RLDS}=\frac{L}{D_{2}}$$

where $D_{2}$ denotes the diameter of the stalk and L represents the length of the stalk.

### 2.4. The Proposed SegGrade Algorithm for Morphological Grading of Mushrooms

**Figure 5:**
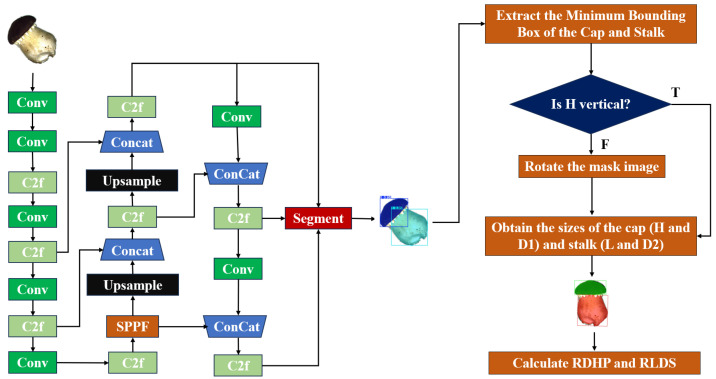
The overall architecture of the proposed SegGrade algorithm.

Based on YOLOv8-seg and OpenCV techniques, the SegGrade algorithm was proposed according to the developed grading criteria. The architecture of the SegGrade algorithm is shown in Figure 5. It consists of two stages: segmentation and grading. In the first stage, the input images are the original images. YOLOv8-seg identifies the caps and stalks of the mushrooms in real time, then outputs segmented images with different colors and labels. In the second stage, the segmented images generated in the first stage are post-processed using the OpenCV algorithm to obtain the images used for grading. Then the mushrooms are graded according to the grading criteria.

#### 2.4.1. The YOLOv8-seg Architecture

**Figure 6:**
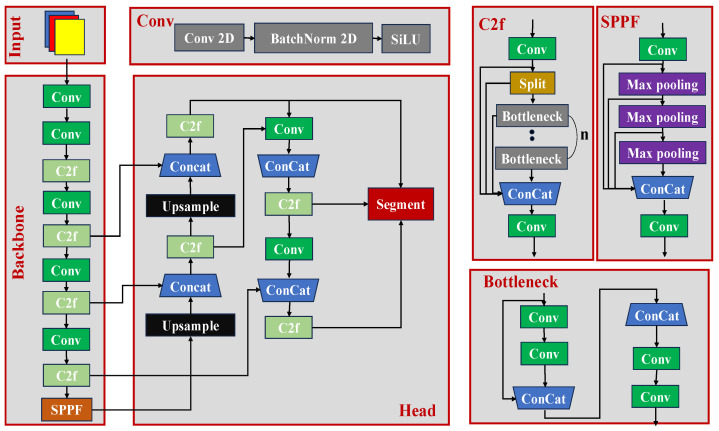
The YOLOv8-seg architecture for segmenting the caps and stalks of the mushrooms.

The first stage of the proposed SegGrade algorithm uses YOLOv8-seg to identify mushrooms and obtain segmented images. The network architecture of YOLOv8-seg is shown in Figure 6. YOLOv8-seg consists of three parts, including the input part, the backbone part, and the head part (including the segmentation output part) [28].

The input layer initiates the entire network process. The input part is responsible for receiving images and performing pre-processing operations, including normalization and resizing. The input part was designed to facilitate subsequent feature extraction and processing.

The backbone part is the feature extraction part. YOLOv8-seg basically follows the backbone of YOLOv5, but some modules were modified to improve the network performance. To make the model more lightweight, a 3 × 3 convolution is used instead of the initial 6 × 6 convolution block. In addition, the C2f module is used instead of the C3 module. It combines the features of the C3 and ELAN modules and allows for richer gradient flow during the backpropagation process. The backbone part is used to extract multi-scale feature maps from the input image and passes them to the neck.

The head part includes the neck and segmentation output. It is responsible for fusing the different scales of the feature maps output from the backbone network and generating detection results and segmentation mask images. YOLOv8-seg uses a path aggregation network (PANet) and a feature pyramid network (FPN), which can fully mobilize the features of all scales, and it is very effective in detecting both large and small targets [29].

#### 2.4.2. Post-Processing Based on OpenCV

The second stage of the proposed SegGrade algorithm is the post-processing of the segmented images output from YOLOv8-seg, which required the development of a grading algorithm suitable for the grading criteria based on OpenCV.

**Figure 7:**
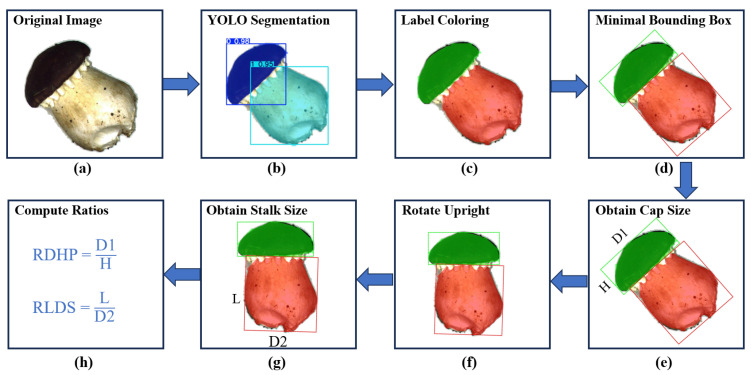
The OpenCV-based post-processing framework for mushroom grading. (**a**) Original image. (**b**) YOLO segmentation. (**c**) Label coloring. (**d**) Minimal bounding box. (**e**) Obtain cap size. (**f**) Rotate upright. (**g**) Obtain stalk size. (**h**) Compute ratios.

The post-processing framework based on OpenCV is shown in Figure 7. Figure 7a–h fully demonstrate the post-processing process. The input image is first fed into the YOLOv8-Seg segmentation model to extract accurate masks of the mushroom cap and the mushroom stalk. These masks are then passed to OpenCV for processing, where the mushroom cap area is rendered green and the mushroom stalk area is rendered red to facilitate subsequent morphological analysis. For each mask, its minimum bounding rectangle (the smallest axis-aligned rectangular box that surrounds all foreground pixels) is extracted. Since the “length” (L), which refers to its size along the growth direction, and “diameter” (D_2_), which refers to its width perpendicular to the growth direction, of the mushroom stalk may be in any direction in the image and the two cannot be directly distinguished numerically, additional reference information is needed. It was observed in the experiment that the diameter (D_1_) of a mushroom cap was always greater than its height (H). Its orientation could be inferred from these values, thereby assisting in determining the geometric properties of the stalk. Therefore, the minimum bounding rectangle of the mushroom cap is first analyzed to extract its height (H) and diameter (D_1_), and the image is rotated accordingly to align the height (H) with the vertical direction in the image. After the rotation is completed, the minimum circumscribed rectangle of the mushroom stalk is analyzed. The side closest to the vertical direction is taken as the actual length (L), and the direction perpendicular to this side is taken as the diameter (D_2_). Finally, the RDHP and RLDS are calculated according to Formulas (1) and (2) to determine the grade of each mushroom.

### 2.5. Evaluation Criteria

In this study, the proposed SegGrade algorithm and the automatic grading system needed to be evaluated separately using different metrics to verify their feasibility. To evaluate the segmentation performance, params (parameters), inference time, and floating-point operations (FLOPs) were selected as evaluation metrics for the segmentation efficiency of the algorithm, while precision, recall, the F1-score, the mean intersection over union (MioU), and the mean pixel accuracy (MPA) were chosen as evaluation metrics for the segmentation accuracy of the algorithm. Params were used to evaluate the memory resources occupied by the algorithm; inference time was used to evaluate the detection speed of the algorithm, which is an extremely important parameter in real-time tasks; and FLOPs represented the amount of computation, which was utilized to assess the computational complexity of the algorithm. In addition, the loss function of the algorithm is an important index that was used to evaluate the performance of the algorithm [30]. A lower loss value indicated that the predicted result was closer to the actual value, reflecting better model robustness. The loss function of YOLOv8-seg consists of three parts: box_loss, seg_loss, and cls_loss. The above metrics can be calculated as follows:

(3) $$\mathrm{Precision}=\frac{\mathrm{TP}}{\left(\mathrm{TP}+\mathrm{FP}\right)}$$

(4) $$\mathrm{Recall}=\frac{\mathrm{TP}}{\left(\mathrm{TP}+\mathrm{FN}\right)}$$

(5) $$F1-\mathrm{score}=2\times \mathrm{Precision}\times \frac{\mathrm{Recall}}{\left(\mathrm{Precision}+\mathrm{Recall}\right)}$$

(6) $$\mathrm{MIoU}=\frac{1}{k+1}\sum_{i=0}^{k}\frac{p_{\mathrm{ii}}}{\sum_{j=0}^{k}p_{\mathrm{ij}}+\sum_{j=0}^{k}p_{\mathrm{ji}}-p_{\mathrm{ii}}}$$

(7) $$\mathrm{PA}=\frac{\sum_{i=0}^{k}p_{\mathrm{ii}}}{\sum_{i=0}^{k}\sum_{j=0}^{k}p_{\mathrm{ij}}}$$

(8) $$\mathrm{MPA}=\frac{1}{k+1}\sum_{i=0}^{k}\frac{p_{\mathrm{ii}}}{\sum_{j=0}^{k}p_{\mathrm{ij}}}$$

(9) $$\mathrm{loss}=\mathrm{bo}x_{\_}\mathrm{loss}+\mathrm{seg}\_\mathrm{loss}+\mathrm{cls}\_\mathrm{loss}$$

where true positive (TP) denotes a sample that is correct and recognized as correct, false positive (FP) denotes a sample that is incorrect but recognized as correct, and false negative (FN) denotes a sample that is correct but recognized as incorrect. k represents the count of the target object categories, k+1 is the total count of the categories including the background class, $p_{\mathrm{ii}}$ means that the pixel belongs to category *i* and is correctly classified as category *i*, $p_{\mathrm{ij}}$ means that the pixel belongs to category *i* but is incorrectly classified as category *j*, and $p_{\mathrm{ji}}$ means that the pixel belongs to category *j* but is incorrectly classified as category *i*.

To evaluate the performance of the SegGrade algorithm and the overall grading system, accuracy was selected as the evaluation index. It indicates the average of the correct recognition rates for each grade of the mushroom. Assuming the total numbers of mushrooms in the first, second, and third grades are *N*1, *N*2, and *N*3 and the numbers of correctly recognized mushrooms are *n*1, *n*2, and *n*3, the accuracy is calculated using the following formula:

(10) $$\mathrm{Accuracy}=\frac{1}{3}\times \left(\frac{n1}{N1}+\frac{n2}{N2}+\frac{n3}{N3}\right)$$

### 2.6. Experimental Configuration and Model Parameters

To ensure the fairness of the experiments, all algorithms in this study were tested using the same equipment. The experiments were executed in Windows 11 (64-bit) with an Intel Core i9-14900K central processing unit (CPU) operating at 6.00 GHz and an NVIDIA GeForce RTX 4080 16G graphics processing unit (GPU). The algorithms were implemented using Python v3.9 and the PyTorch v2.1.2 framework, and the integrated development environment (IDE) was Visual Studio Code v1.102 (VS Code).

Six state-of-the-art (SORT) segmentation algorithms, including U-net [31], DeeplabV3+ [32], PSPnet [33], HRNet [34], YOLOv5-seg, YOLOv8-seg, YOLOv10-seg, and YOLO11-seg, were selected to be applied to recognize the caps and stalks of the mushrooms. After completing the above dataset preparation, algorithm construction, and experimental configuration, the parameters of each model were finally determined after tuning the eight algorithms. The key parameters are shown in Table 3.

## 3. Results

### 3.1. Validation of the Segmentation Algorithm

#### 3.1.1. Segmentation Performance Analysis of Different Segmentation Algorithms

To achieve accurate and efficient mushroom segmentation, this study adopted lightweight models from the YOLO series. YOLOv5-seg uses the small (s) model, while YOLOv8-seg, YOLOv10-seg, and YOLO11-seg utilize the lightweight nano (n) models from the YOLO series. By comparing the performances of the different algorithms, the most suitable algorithm for segmenting the mushrooms was identified.

The comparison of algorithm accuracy in segmenting the cap and stalk portions of mushrooms, as demonstrated in Table 4, revealed that YOLOv8-seg exhibited the best detection performance. The precision and recall of YOLOv8-seg both reached 99.85%, with an MIoU of 99.70%, which indicates its exceptional accuracy and reliability in identifying cap and stalk instances. YOLOv10-seg and YOLO11-seg also showed excellent performance, with both achieving an F1-score of 99.70%. YOLOv10-seg reached an MIoU of 99.60%, and YOLO11-seg achieved 99.41%, slightly lower than YOLOv8-seg but still strong and reliable segmentation results. YOLOv5-seg demonstrated impressive overall accuracy as well, with an F1-score of 99.69% and a precision of 99.71%, showing that even with a smaller model size, it maintains high segmentation performance and is suitable for real-time applications. The MPA (99.46%) and recall (99.46%) scores of the U-net algorithm were among the highest among the traditional segmentation models, although they were still lower than those of the YOLO-based methods. DeeplabV3+ had a balanced performance in terms of the F1-score (99.00%) and MPA (99.22%) but showed poor results in terms of the MIoU (98.04%), which limited its effectiveness in segmenting the caps and stalks of mushrooms. HRNet performed consistently in the segmentation task, with an F1-score of 99.25%, indicating a good balance between precision and recall. Among all of the algorithms, PSPnet had the lowest metrics, indicating that this algorithm performs relatively poorly in the task of recognizing the caps and stalks of mushrooms.

The mushroom segmentation efficiency of each algorithm was compared, and the results show that the YOLO series algorithms exhibited a clear advantage, as shown in Table 5. YOLOv8-seg and YOLO11-seg had params of 3.26 × 10^6^ and 2.83 × 10^6^. The FLOPs were 12.00 and 10.20, and the inference times reached 13.23 ms and 14.32 ms. The two algorithms had low parameters, fast inference speeds, and small computation values, which were very suitable for real-time and efficient detection tasks. Similarly, YOLOv5-seg and YOLOv10-seg also maintained lightweight structures with params of 2.76 × 10^6^ and 2.84 × 10^6^ and fast inference times of 15.10 ms and 14.26 ms, respectively, showing strong efficiency. In addition, U-net and PSPNet had significantly higher parameters and FLOPs, resulting in longer inference times of 92.68 ms and 103.13 ms. Although these two algorithms were relatively strong with respect to segmentation accuracy, they were far inferior to YOLOv8-seg with respect to inference efficiency. DeeplabV3+ and HRNet achieved a certain balance between efficiency and accuracy. Compared with U-net and PSPNet, DeeplabV3+ (5.81 × 10^7^) and HRNet (9.64 × 10^7^) had relatively small params, with inference times of 15.79 ms and 50.22 ms, respectively, but their overall performance was still inferior to that of the YOLO series. Among all of the algorithms, YOLOv8-seg combined high accuracy and fast inference capability and was suitable for real-time application.

#### 3.1.2. Validation of the Mushroom Segmentation Algorithm

**Figure 8:**
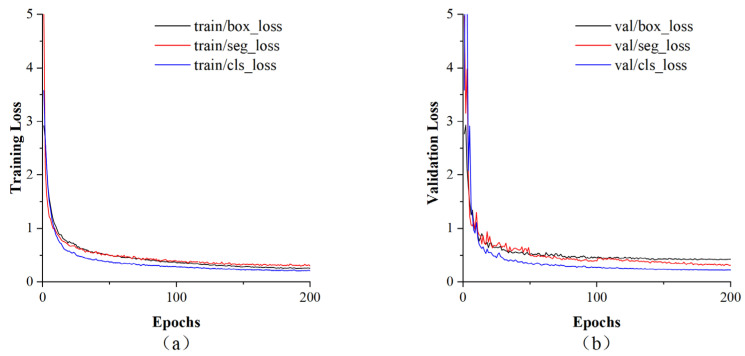
The loss curves of YOLOv8-seg for the mushroom segmentation. (**a**) Training loss curve. (**b**) Validation loss curve.

**Figure 9:**
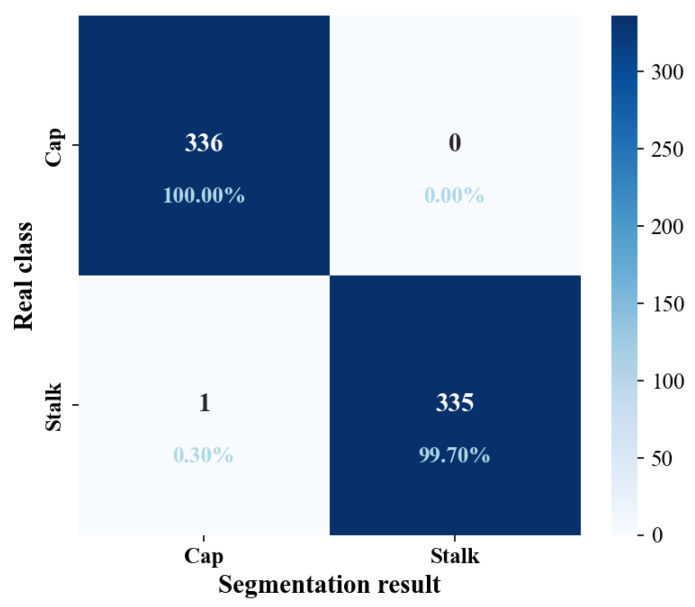
The confusion matrix of YOLOv8-seg for the mushroom segmentation.

This work used YOLOv8-seg to detect and segment mushroom caps, and it was necessary to evaluate the overall performance of the algorithm. The training and validation loss curves for YOLOv8-seg are shown in Figure 8. They show loss trends during training and were used to evaluate the performance of the algorithm at different stages. The training and validation loss curves for YOLOv8-seg dropped to about 0.5 after 20 epochs and finally stabilized after about 150 epochs. This shows that the algorithm achieved a good fitting effect for localization, segmentation, and classification. A confusion matrix of the YOLOv8-seg mushroom segmentation is shown in Figure 9, which intuitively presents the grading results and facilitates misclassification analysis. The diagonal elements of the matrix represent the number of correctly classified samples, while the off-diagonal elements represent the number of misclassified samples [35]. YOLOv8-seg successfully identified 336 caps without misclassifying any as stalks, giving a segmentation accuracy of 100% for the caps. YOLOv8-seg correctly identified 335 stalks, with only 1 stalk being misclassified as a cap, giving a segmentation accuracy of 99.70% for the stalks. Overall, YOLOv8-seg showed sufficient accuracy in segmenting the caps and stalks of mushrooms.

### 3.2. Validation of the Proposed SegGrade Algorithm for Mushrooms

**Figure 10:**
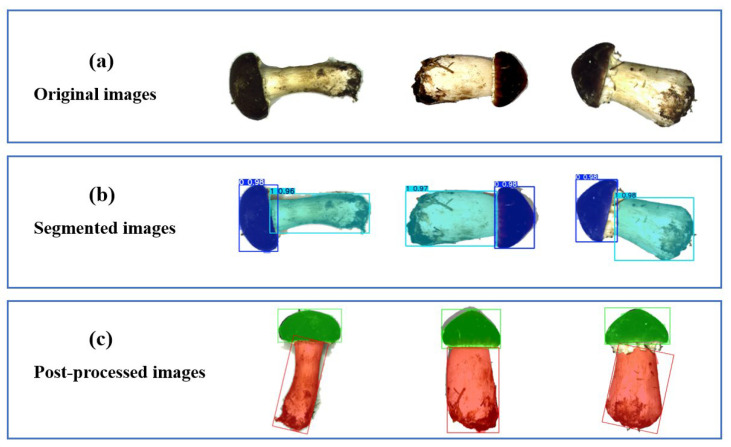
Representative examples of the proposed SegGrade algorithm for mushroom identification in a real conveyor belt scenario. (**a**) Original images. (**b**) Segmented images. (**c**) Post-processed images.

The test results for the SegGrade algorithm in an actual conveyor belt scenario are shown in Table 6. A performance experiment using 50 randomly selected mushrooms from each of the three grades (150 in total) showed that the proposed SegGrade algorithm can achieve an accuracy of 94.67%, which indicates that the proposed SegGrade algorithm has high grading accuracy in this task. Representative examples of grading the stalk and cap using the SegGrade algorithm are shown in Figure 10. Specifically, the process consists of capturing original images of mushrooms on a conveyor belt, using YOLOv8-seg to obtain segmented images, and using OpenCV to grade them. The SegGrade algorithm, which integrates segmentation and post-processing, enhances the accuracy of cap and stalk region extraction, thereby improving grading precision.

### 3.3. Motion Trajectory Analysis of the Mushrooms

To accurately and non-destructively achieve continuous air-blown sorting of the mushrooms, it was necessary to determine whether the mushrooms could be blown off at a given air pressure. In addition, analyzing their falling positions was essential for designing the size and position of the collection box. Therefore, it was particularly important to study the movement trajectories of air-blown mushrooms.

Before blowing, the mushrooms are traveling at the same forward horizontal velocity ($V_{x}$) as the conveyor belt, and there is no external force. Whether the mushrooms can be displaced by the air-blown force depends on whether this force exceeds the frictional force exerted by the conveyor belt. The equivalent relationship between these forces can be expressed as follows:

(11) PS=μmg

(12) $$S=\pi r^{2}$$

where P is the pressure of the air pump, S is the cross-sectional area of the cylindrical air tube, r is the radius of the cross-section of the air tube, μ is the coefficient of friction of the conveyor belt, m is the mass of the mushroom, and g is the acceleration of gravity.

In this equation, the air pressure (P) of the air pump is a fixed value of 0.80 MPa and r is 4 mm. When π is taken to be 3.14, S is 50.24 mm^2^, so the air pressure is 40.19 N. The coefficient of friction of the conveyor belt (μ) is usually 0.10, and g is usually taken to be 9.80. The two are only comparable if m is 41.01 kg, while the average mass of a mushroom is in the region of 30 g, so the air must be able to remove a mushroom.

**Figure 11:**
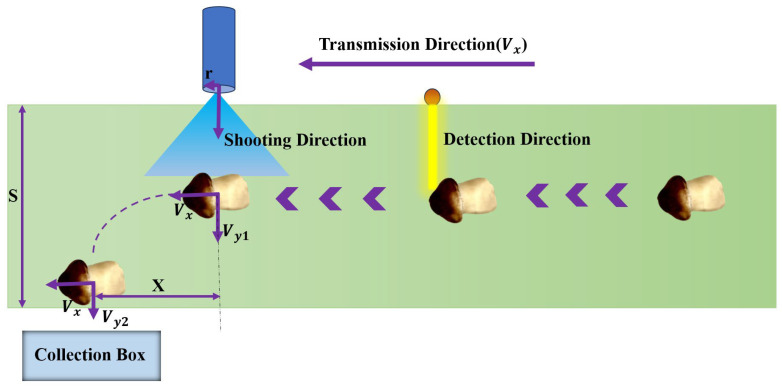
The motion trajectory of a mushroom.

The trajectory analysis of the mushrooms can be divided into two cases, which are subject to an air-blowing force and only friction force. The air-blowing process is shown in Figure 11. Assuming the blowing time (${∆t}_{0}$) is short, there is no positional change for the mushroom during the moment of blowing. Since the air-blowing force greatly exceeds the friction of the conveyor belt, the vertical velocity of the mushroom ($V_{y1}$) after blowing can be obtained from the impulse formula, as shown in Equation (13):

(13) $$V_{y1}=\frac{P{∆t}_{0}+mV_{y0}}{m}$$

where $V_{y0}$ is the initial vertical velocity of the mushroom.

When the blowing is over, the mushroom is only subjected to the friction of the conveyor belt, and the acceleration of the mushroom at this time (a) can be calculated by Equation (14):

(14) $$a=\frac{\mu \mathrm{mg}}{m}$$

Assuming that the initial position of the mushroom is located in the middle of the conveyor belt, after blowing the mushroom, without any external force in the horizontal direction ($V_{x}$), the only influence is the friction of the conveyor belt. The velocity in the vertical direction ($V_{y2}$) can be calculated by Equation (15), the falling time (${∆t}_{1}$) can be calculated by Equation (16), and the horizontal displacement of the mushroom (X) can be calculated by Equation (17):

(15) $$V_{y2}=\sqrt{2a*\frac{S}{2}+{V_{y1}}^{2}}$$

(16) $$∆t1=\frac{V_{y2}-V_{y1}}{a}$$

(17) $$X=∆t1V_{x}$$

where S is the width of the conveyor belt.

In the above derivation, the mass of the mushroom is the only variable; the other variables are already quantitative after the construction of the system is completed. Therefore, based on the minimum and maximum masses of the mushrooms, it was possible to determine that the horizontal displacement of the mushrooms lies roughly between 2.25 cm and 6.00 cm, and thus the sizes and locations of the collection racks were designed to accomplish accurate and non-destructive grading of mushrooms.

### 3.4. Validation of the Proposed Grading System for Mushrooms

In this experiment, a comprehensive evaluation was conducted to determine the grading efficiency and accuracy of the automatic mushroom-grading system. The mushrooms used in this section were the same as those described in Section 3.2. To assess the performance of the automatic grading system under consistent external conditions, three repeated experiments were conducted. The grading results are presented in Table 7. The accuracy rates of the three trials were 83.33%, 83.33%, and 75.33%, yielding an average accuracy of 80.66%. Notably, no physical damage to the *Stropharia rugoso-annulata* mushrooms occurred during the grading process. Variations in accuracy across the three trials are reflected in the standard error values, which highlight the system’s grading stability under controlled conditions. The slight differences in accuracy can be attributed to the mechanical responses during the experiments. These findings underscore the importance of further improving system robustness to minimize variability and ensure consistently high accuracy across repeated trials.

The average grading accuracies for the first-grade, second-grade, and third-grade mushrooms were 90.67%, 82.67%, and 68.67%, respectively. The results indicate that the grading system achieved the highest accuracy for first-grade mushrooms, while the lowest accuracy was observed for third-grade mushrooms. This discrepancy is primarily attributed to the varying mechanical responses of mushrooms with different morphological characteristics under air-blowing conditions. Specifically, third-grade mushrooms were more prone to lateral displacement due to their elongated and slender shapes, which led to a higher rate of misclassification. In contrast, first-grade mushrooms, characterized by a thicker and shorter structure, experienced a more uniform force distribution under airflow due to a larger contact area, resulting in significantly better grading performance. To address this issue, future research will focus on optimizing the air nozzle by introducing dynamically adjustable airflow to accommodate the structural differences among the mushroom grades. Additionally, the grading efficiency was evaluated by placing 50 mushrooms on the vibrating feeder and counting the number of mushrooms successfully sorted into the collection boxes within one minute. The system demonstrated an average grading capacity of approximately 20 mushrooms per minute, which is considered moderate and sufficient to meet practical application requirements.

## 4. Discussion

The SegGrade algorithm integrates YOLOv8-seg with OpenCV-based post-processing, establishing a novel two-stage grading framework. Based on this SegGrade algorithm, an automatic air-blown grading system was developed. The YOLOv8-seg component achieved 99.50% segmentation accuracy with an inference speed of 13.23 ms, ensuring real-time operation while maintaining grading precision. Unlike existing studies that mostly use subjective visual standards or single size parameters, the SegGrade algorithm innovatively proposes two quantitative indicators (the RDHP and RLDS). This dual-indicator evaluation system realized objective quantitative grading of mushroom appearance quality for the first time. Furthermore, the air-blown grading system, leveraging a dynamic adjustment mechanism to match mushroom morphology, achieved a practical grading accuracy of 80.66% and a throughput of 20 mushrooms per minute without causing damage. Compared with mechanical systems that risk physical damage, this innovative non-contact approach marks a significant advancement in automated mushroom-grading technology.

One of the main advantages of the proposed automatic grading system is its ability to effectively separate the stalks and caps of mushrooms for fast and accurate grading. Previous grading algorithms based on computer vision or machine learning often struggle to extract images accurately and have slow detection speeds, so they cannot be applied to real-time tasks [36,37,38]. Most studies used deep learning methods to classify crops, but the feature extraction process of the deep learning models lacked interpretability, making it difficult to accurately adjust the grading criteria. Fan et al. [39] used a CNN-based deep learning architecture and a cost-effective computer vision module to detect defective apples. Hu et al. [40] used a support vector machine (SVM) and a convolutional neural network (CNN) to classify early bruises of apples in real time. Mukasa et al. [41] used the YOLOv5 model and online gravity-fed systems to perform real-time sorting of watermelon seeds by purity. In the preliminary study, although quantitative grading indexes were proposed, deep learning was still used to randomly acquire the appearance features of mushrooms, and the study focused on the lightweight treatment of models [12]. Based on this research, this study introduced quantitative grading indicators and proposed the SegGrade grading algorithm to achieve more accurate and scientific grading of the appearance features. The SegGrade algorithm proposed in this experiment not only focuses on the morphological appearance of mushrooms but also relies on quantitative grading metrics for size grading. During the segmentation stage, the algorithm extracts and processes the cap and stalk, ensuring that damaged specimens are excluded from the air-blown grading process. Although grading was implemented in a simple conveyor belt environment, the SegGrade algorithm is highly versatile and can automatically grade mushrooms in various scenarios by enriching the training dataset. The grading accuracy of the SegGrade algorithm is sufficient, and future efforts will focus on improving its grading speed to enhance the overall efficiency of the automatic system.

The proposed automatic mushroom-grading system aims to ensure non-destructive, fast, and accurate grading. Air-blown grading is also a feature and advantage of this study. Wang et al. [42] designed a mechanical clamp assembly to grade walnuts with surface defects and achieved an average loss rate of 6.50%. Zhang et al. [43] used a sorter with an aluminum paddle to sort apples, and only 55% of the apples did not show any bruises after sorting or grading. Such mechanical parts are extremely likely to damage the appearance of mushrooms, which would be very unfavorable when selling the mushrooms, thus necessitating the use of the non-contact air-blown grading method. In addition, mechanical structures require time to move mechanical parts, whereas air-blown grading is very fast, with variable airflow directions and intensities, and is capable of sorting mushrooms of different appearances. In this study, the grading accuracy of the SegGrade algorithm reached 94.67%. However, the overall grading accuracy of the automatic system was 80.66%. This grading error was mainly due to differences in mushroom shape and size. Third-grade mushrooms are more likely to be excessively displaced during the grading process due to their slender shapes, resulting in grading errors. Current problems and possible future improvements to the automatic air-blown grading system are as follows: (1) In terms of the feeding stage, although the structure of the vibrating disk can output individual mushrooms, the increase in frequency reduces the spacing between neighboring mushrooms. However, air-blown grading of individual mushrooms takes time, which limits the frequency of vibration. In future work, an electronically controlled baffle structure will be added to ensure that the vibrating disk automatically outputs individually spaced mushrooms. (2) In terms of air-blown grading, to address the variations in the mechanical responses among mushrooms of different morphologies, the air pressure will be automatically adjusted according to the different grades of mushrooms so that there will be suitable air pressure to complete the grading for all mushrooms. (3) The coordinated timing of the sensors and actuators will be optimized to reduce errors caused by response delays.

In summary, the proposed automatic grading system has great potential to improve the automation of mushroom production and marks a valuable step towards the modernization of agriculture. However, the system must be further optimized to meet the stringent requirements of large-scale commercial applications. In the future, further development of the system’s hardware and algorithms will focus on the issues raised in this study to ensure improved grading accuracy and efficiency.

## 5. Conclusions

In this study, the SegGrade algorithm was proposed, and an automatic system for morphological grading of edible mushrooms was designed based on this algorithm. It enables fast, non-destructive, and accurate grading of different grades of mushrooms. In this experiment, after comparing several segmentation algorithms, YOLOv8-seg was chosen to automatically identify the stalk and cap parts of mushrooms. YOLOv8-seg achieved real-time segmentation while maintaining 99.50% accuracy. An OpenCV-based grading algorithm was developed based on the obtained segmented images. Experiments were carried out in practical application scenarios, and the results show that the recognition accuracy of the proposed SegGrade algorithm can reach 94.67%, which is sufficient to meet practical requirements. In addition, the trajectories of the mushrooms were analyzed, and the results show that the horizontal displacement of the mushrooms after blowing ranged from 2.25 to 6.00 cm. The sizes and locations of the collection boxes were designed accordingly. In full-scale testing, the system maintained 80.66% grading accuracy at 20 units per minute while preserving structural integrity. However, there is still room for improvement in the automatic mushroom-grading system, and optimization of the system’s structure will be given top priority in the future to further improve the grading efficiency and accuracy of the entire automatic system, with the goal of achieving fully automated production.

## Figures and Tables

**Table 1:** The dataset construction for the different mushroom grades.

Dataset	First Grade	Second Grade	Third Grade	Total
Training Set	336	336	336	1008
Validation Set	112	112	112	336
Test Set	112	112	112	336
Total	560	560	560	1680

**Table 2:** The proposed grading criteria for the mushrooms in conjunction with the local grading status in Pinggu District.

Grade	RDHP	RLDS	Grading Rule
First Grade	1.5 ~ 2.5	0 ~ 1.5	Grading is based on RDHP and RLDS ranges; RLDS prevails in conflicts.
Second Grade	1.0 ~ 1.5	1.5 ~ 2.5	
Third Grade	0 ~ 1.0	>2.5	

**Table 3:** The key parameters of the evaluated models.

Model	Image Size	Batch Size	Learning Rate
U-net	512 × 512 × 3	8	1 × 10^−4^
DeeplabV3+	512 × 512 × 3	8	1 × 10^−4^
PSPnet	512 × 512 × 3	8	1 × 10^−4^
HRNet	512 × 512 × 3	8	1 × 10^−4^
YOLOv5-seg	512 × 512 × 3	16	4 × 10^−4^
YOLOv8-seg	512 × 512 × 3	16	4 × 10^−4^
YOLOv10-seg	512 × 512 × 3	16	4 × 10^−4^
YOLO11-seg	512 × 512 × 3	16	4 × 10^−4^

**Table 4:** Comparison of mushroom segmentation accuracy of different segmentation algorithms.

Model	F1-Score (%)	MPA (%)	MIoU (%)	Precision (%)	Recall (%)
U-net	99.33	99.46	98.67	99.20	99.46
DeeplabV3+	99.00	99.22	98.04	98.79	99.22
PSPnet	95.22	94.43	90.97	96.03	94.43
HRNet	99.25	99.45	98.50	99.05	99.45
YOLOv5-seg	99.69	99.39	99.40	99.71	99.68
YOLOv8-seg	**99.85**	**99.50**	**99.70**	**99.85**	**99.85**
YOLOv10-seg	99.70	99.40	99.60	99.66	99.75
YOLO11-seg	99.70	99.38	99.41	99.70	99.70

The bold text indicates the optimal value for each metric.

**Table 5:** Comparison of mushroom segmentation efficiency of different segmentation algorithms.

Model	Params	Inference Time (MS)	FLOPs (G)
U-net	2.49 × 10^7^	92.68	226.15
DeeplabV3+	5.81 × 10^7^	15.79	26.44
PSPnet	4.91 × 10^7^	103.13	36.02
HRNet	9.64 × 10^7^	50.22	18.74
YOLOv5-seg	2.76 × 10^6^	15.10	11.00
YOLOv8-seg	3.26 × 10^6^	**13.23**	12.00
YOLOv10-seg	2.84 × 10^6^	14.26	11.70
YOLO11-seg	**2.83 × 10^6^**	14.32	**10.20**

The bold text indicates the optimal value for each metric.

**Table 6:** The test results of the proposed SegGrade algorithm for mushrooms in a real conveyor belt scenario.

Grade	Total Number	Correct Number	Correct Rate (%)	Accuracy (%)
First grade	50	49	98.00	
Second grade	50	47	94.00	94.67
Third grade	50	46	92.00	

**Table 7:** The grading system test results from a real experiment.

Group	Grade	Total Number	Correct Number	Correct Rate (%)	Accuracy (%) ± Error
Group 1	First	50	45	90.00	83.33 ± 2.5
	Second	50	42	84.00	83.33 ± 3.0
	Third	50	38	76.00	83.33 ± 4.0
Group 2	First	50	48	96.00	83.33±1.5
	Second	50	42	84.00	83.33 ± 2.0
	Third	50	35	70.00	83.33 ± 3.5
Group 3	First	50	43	86.00	75.33 ± 2.0
	Second	50	40	80.00	75.33 ± 2.5
	Third	50	30	60.00	75.33 ± 3.0

## Data Availability

Data will be made available on request.

## Supplementary Materials

The following supporting information can be downloaded at: https://www.mdpi.com/article/10.3390/s25144482/s1.

## Abbreviations

The following abbreviations are used in this manuscript:

YOLOv5	You Only Look Once Version 5
FPS	Frames Per Second
mAP	Mean Average Precision
MS	Millisecond
ASA-FPN	Shuffled Adaptive Spatial Feature Pyramid Network
MYOLO	Mushroom You Only Look Once
KG	Kilogram
PC	Personal Computer
USB	Universal Serial Bus
CM	Centimeter
RDHP	Ratio of Diameter to Height of the Cap
RLDS	Ratio of Length to Diameter of the Stalk
PANet	Path Aggregation Network
FPN	Feature Pyramid Network
FLOPs	Floating-Point Operations
MioU	Mean Intersection Over Union
MPA	Mean Pixel Accuracy
TP	True Positive
FP	False Positive
FN	False Negative
CPU	Central Processing Unit
GPU	Graphics Processing Unit
IDE	Integrated Development Environment
VS Code	Visual Studio Code
SORT	State-Of-The-Art
MM	Millimeter
