# Supplementary figures and images for: Automated Discrimination of Appearance Quality Grade of Mushroom (Stropharia rugoso-annulata) Using Computer Vision-Based Air-Blown System
Source: Sensors (Basel). 2025 Jul 18;25(14):4482. doi: 10.3390/s25144482 (PMC12299747; doi:10.3390/s25144482)

## Slide 1
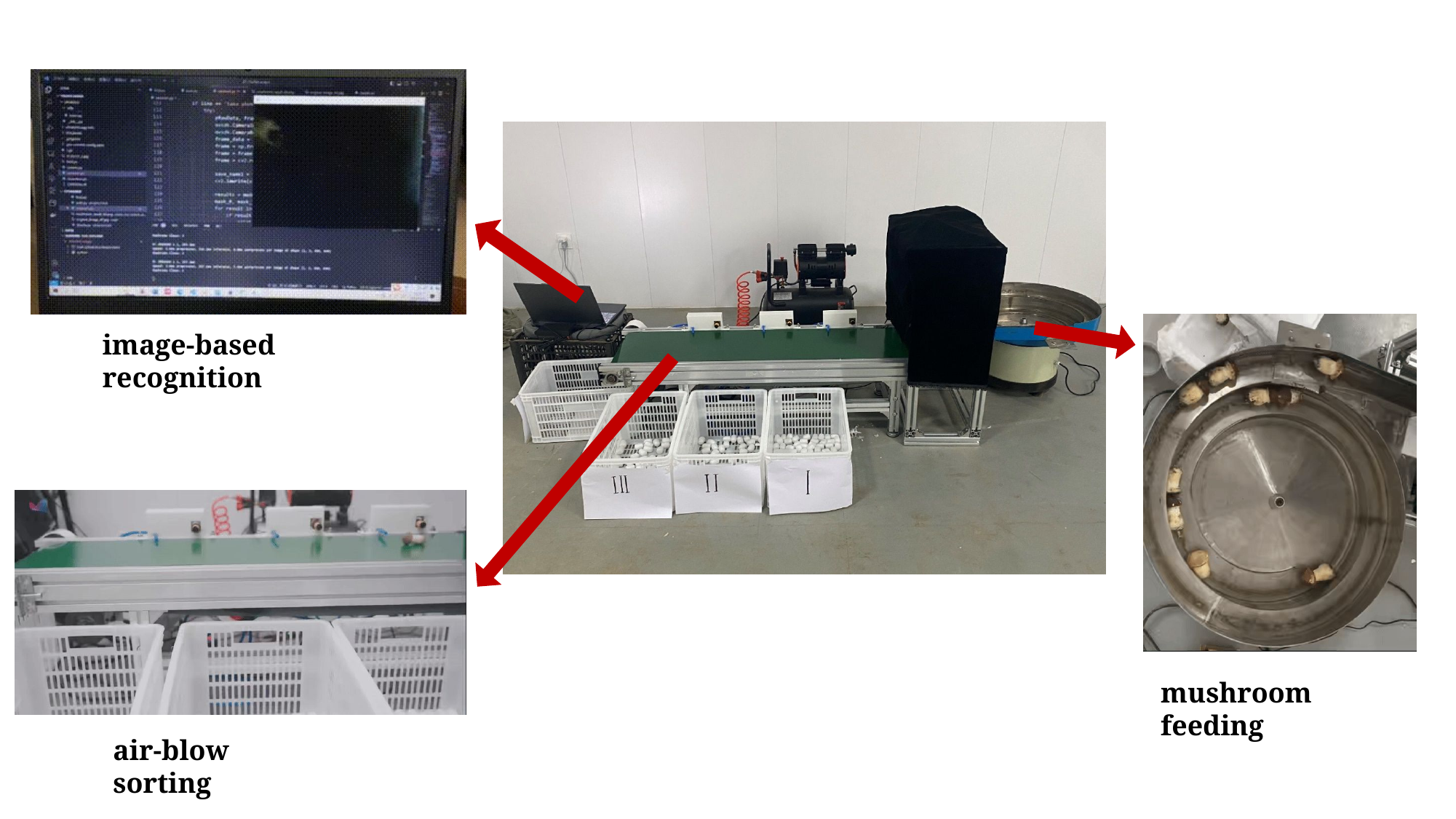

image-based recognition
mushroom feeding
air-blow sorting

Supplement: Supplementary file 1 [file sensors-25-04482-s001.zip › sensors-3709333-supplementary.pptx]
